# Genome Sequences of Two Pandoraea pnomenusa Isolates Recovered 11 Months Apart from a Cystic Fibrosis Patient

**DOI:** 10.1128/genomeA.01389-14

**Published:** 2015-02-05

**Authors:** Robson Ee, Mark Ambrose, James Lazenby, Paul Williams, Kok-Gan Chan, Louise Roddam

**Affiliations:** a Division of Genetics and Molecular Biology, Institute of Biological Sciences, Faculty of Science, University of Malaya, Kuala Lumpur, Malaysia; b School of Medicine, University of Tasmania, Tasmania, Australia; c Centre for Biomolecular Sciences and School of Life Sciences, University of Nottingham, Nottingham, United Kingdom

## Abstract

Pandoraea is an emerging respiratory pathogen capable of causing chronic lung infections in people with cystic fibrosis (CF), but the clinical significance of this infection is ambiguous. We have sequenced and annotated the genomes of two multidrug-resistant Pandoraea pnomenusa isolates recovered 11 months apart from the same CF patient.

## GENOME ANNOUNCEMENT

Pandoraea species have been isolated from cystic fibrosis (CF) and non-CF patients with respiratory tract infections (1), yet the role of these organisms in respiratory disease is currently unclear. Complicating matters is the fact that Pandoraea differentiation from closely related bacteria, like Burkholderia cenocepacia complex and Ralstonia spp. (2), is difficult, and Pandoraea infection is also frequently associated with cocolonization by other respiratory pathogens, most notably Pseudomonas aeruginosa.

In CF patients, Pandoraea is being increasingly isolated from the lungs and has the potential to cause chronic airway infections (2–4) associated with decreased lung function and increased frequency of pulmonary exacerbation (4–8). In addition, several Pandoraea infections in CF patients have progressed to bacteremia, in which the majority of cases appear to have been caused by Pandoraea pnomenusa (9–11); this in turn suggests an increased potential of this organism for invasive disease (11). Furthermore, there are concerns regarding the high antimicrobial resistance of Pandoraea (4, 8, 10, 12) and patient-to-patient transmission (2, 4), leading some centers to strongly recommend patient segregation (4, 5, 13).

P. pnomenusa strain 6399 was isolated from a sputum sample from a CF patient and identified by matrix-assisted laser desorption ionization–time of flight mass spectrometry (MALDI-TOF MS) and 16S rRNA sequencing. Strain 7641 was isolated from the same patient 11 months later, which suggested that this patient had a chronic lung infection. Both isolates have multiantibacterial resistance properties and proved to be sensitive to imipenem and co-trimoxazole. Total bacterial genomic DNA was extracted and purified using the AxyPrep DNA extraction kit (Axygen Biosciences, USA) and subsequently subjected to whole-genome sequencing on a MiSeq desktop sequencer (Illumina, Inc., USA). The paired-end reads were assembled with CLC Genomics Workbench version 7.5. The draft genome of strain 6399 is 5,574,597 bp with a 62.9% G+C content, while that of strain 7641 is 5,577,534 bp with a 62.8% G+C content.

A total of 60 and 61 tRNAs were predicted for strains 6399 and 7641, respectively, using ARAGORN (14), and a complete set of rRNA operons consisting of 5S, 16S, and 23S rRNA was predicted in both genomes using RNAmmer (15). The predicted 16S rRNA sequences were submitted to the EzTaxon-e database, which reconfirmed the identity of both strains as P. pnomenusa, with a 99.73% pairwise similarity (16). Subsequently, the Rapid Annotations using Subsystems Technology (RAST) pipeline (17) was used to predict and annotate open reading frames (ORFs). The strain 6399 and 7641 genomes comprise 5,048 and 5,047 protein-coding ORFs, respectively. The genomes of the two isolates are very similar with that of strain 6399, having one extra gene that encodes a predicted phage tail protein. In comparison to the genome sequence of the environmental P. pnomenusa strain RB38 (18), these clinical isolates have 152 unique genes (mostly virulence genes) and appear to be missing 87 genes.

In the genomes of both clinical isolates, 130 genes responsible for virulence, disease, and defense were identified, and predictably, many of these genes are associated with antimicrobial resistance mechanisms (105 ORFs). The annotated genomes of these two clinical P. pnomenusa strains represent valuable tools for studying the antibiotic resistance mechanisms and virulence potential of this important emerging CF pathogen.

### Nucleotide sequence accession numbers.

These whole-genome shotgun projects have been deposited in DDBJ/EMBL/GenBank under accession numbers JTCR00000000 (strain 6399) and JTCS00000000 (strain 7641). The versions described in this paper are the first versions.
